# High cortisol and the risk of Alzheimer disease: A protocol for systematic review and meta-analysis

**DOI:** 10.1097/MD.0000000000027319

**Published:** 2021-10-01

**Authors:** Zhuang Yao, Bin Liu, Yueyang Wang, Xiaohong Dong

**Affiliations:** a Department of Nursing, Jiamusi College of Heilongjiang University of Chinese Medicine, Heilongjiang, China; b Department of Basic Western Medicine, Jiamusi College of Heilongjiang University of Chinese Medicine, Heilongjiang, China; c Institute of Acupuncture, Heilongjiang University of Chinese Medicine, Heilongjiang, China.

**Keywords:** Alzheimer disease, cortisol, dementia, meta-analysis

## Abstract

**Background:**

Morning cortisol levels have been reported to be elevated among patients with Alzheimer disease (AD). We perform a protocol for systematic review and meta-analysis to assess morning central or peripheral cortisol levels in AD patients as compared with cognitively normal individuals.

**Methods:**

Studies were identified through systematic searches in August 2021 with no restrictions on date and time, language, and publication status using the following bibliographic databases: Embase, Medline, PubMed, Web of Science, Science Direct, and the Cochrane Library. Studies were identified using search terms related to cortisol, Alzheimer disease, and cognitive impairment. The study quality of included papers was evaluated using the “National Institutes of Health (NIH) quality assessment tool for observational cohort and cross-sectional studies.” Statistical analyses were performed using Stata (version 14, StataCorp, College Station, TX).

**Results:**

The findings of this study will be submitted to peer-reviewed journals for publication.

**Conclusion:**

Morning cortisol was elevated in AD patients and may have diagnostic and prognostic values for AD.

## 1 Introduction

Alzheimer disease (AD) is the leading neurodegenerative disease worldwide and a common neurogenic disorder that mainly results in severe memory loss in the elderly (>60 years).^[1,2]^ At present, there is no effective preventive treatment for AD. AD is the most popular cause of dementia in the elderly and is considered a multifactorial disorder.^[3,4]^ AD is largely sporadic although early-onset familial AD can represent up to 5% of the AD cases assessed in memory clinics.

A growing body of evidence suggests that abnormal level of the glucocorticoid hormone is linked with key hallmarks of AD pathogenesis.^[5–7]^ Such observations lend credence to the “glucocorticoid-cascade” hypothesis which suggests that chronic glucocorticoid secretion, as seen after prolonged stress exposure, may contribute to the etiopathogenic pathway leading to cognitive decline and AD in the elderly.^[8]^ Activation of the hypothalamic-pituitary-adrenal (HPA) axis results in the production of glucocorticoids, secreted in the form of cortisol in humans, as a response to stress and for maintaining homeostasis.^[9]^ Hormonal release follows a circadian rhythm that reaches peak levels after 30 to 45 minutes post morning awakening and AD-related HPA axis hyperactivity is most frequently observed as elevated peak cortisol levels in the morning.^[10]^

At the same time, several other studies reported no significant differences in morning cortisol levels between AD patients and cognitively normal controls.^[11,12]^ Despite these contradictory reports, no meta-analysis has yet been conducted to synthesize the available evidence and evaluate the existence or magnitude of the reported associations. Robust comparisons of AD-related cortisol elevation in peripheral samples (e.g., blood, saliva, urine, and hair) and in the cerebrospinal fluid (CSF) may also be important for informing future research and clinical applications. Furthermore, it remains unclear whether morning cortisol hypersecretion occurs in earlier disease stages. Therefore, we perform a protocol for systematic review and meta-analysis to assess morning central or peripheral cortisol levels in AD patients as compared with cognitively normal individuals. We also synthesized longitudinal studies that examined morning hypercortisolism as a risk factor for AD development and cognitive decline among older populations.

## 2 Methods

This meta-analysis was registered at Open Science Framework registries (registration number: 10.17605/OSF.IO/PJX3A) and was conducted in accordance with the guidelines of the Preferred Reporting Items for Systematic Reviews and Meta-Analyses Protocols statement guidelines. Ethics application was not required as this study is based on published trials.

### 2.1 Search strategy

Studies were identified through systematic searches in August 2021 with no restrictions on date and time, language, and publication status using the following bibliographic databases: Embase, Medline, PubMed, Web of Science, Science Direct, and the Cochrane Library. Studies were identified using search terms related to cortisol, Alzheimer disease, and cognitive impairment. Medical Subject Headings (MeSH) terms and free text words were combined for the search. The reference lists of the included studies were also checked for additional studies that were not identified with the database search.

### 2.2 Inclusion and exclusion criteria

Studies were included if they used a cross-sectional, case-control, or cohort design and measured cortisol levels in blood (plasma/serum), saliva, or CSF samples. We only included studies with morning sampling (defined as measurement within the time period from morning awakening to 11:30 a.m.) which reflects circadian peak cortisol levels. Cross-sectional studies were included if they reported cortisol levels in clinically diagnosed AD patients versus a cognitively normal group in an appropriate and extractable form for pooled analysis (e.g., mean levels for each group with standard deviation [SD], standard error [SE], or confidence interval [CI]; or mean differences with SE, CI, or exact *P* value). For inclusion, case-control and cohort studies were required to have morning cortisol levels as exposure and subsequent AD onset or cognitive decline as outcomes. Effect estimates of longitudinal data (e.g., hazard ratios [HR], odds ratios [OR], regression or correlation coefficients) were also required for inclusion.

Studies were excluded if they were conducted in younger populations (<50 years old); did not report sampling time; only presented graphic data that were not extractable even with graphic editing software; did not report specific diagnostic criteria for AD; or focused on populations with comorbidities characterized by chronic hypothalamic-pituitary-adrenal hyperactivity.

### 2.3 Data extraction

We extracted the following information from eligible cross-sectional studies for a quantitative synthesis: clinical diagnosis (AD) with the diagnostic criteria used and sample sizes of patient and control groups, mean morning cortisol values for each group and corresponding SD, SE, or CI, sample type, time of sampling, assay method and units, and population characteristics (country, age, sex, comorbidities, and medications known to confoundedly affect cortisol levels).

Data from eligible case-control and cohort studies were extracted for a qualitative synthesis due to substantial methodological heterogeneity. The data included: population characteristics (country, sample size, age, sex, baseline cognitive status, follow-up years), cortisol measures (sample type, time of sampling, assay method, exposure categories), cognitive outcomes and assessments, statistical methods and effect estimates (based on maximally-adjusted models when possible).

### 2.4 Quality assessment

The study quality of included papers was evaluated using the “National Institutes of Health (NIH) quality assessment tool for observational cohort and cross-sectional studies” (www.nhlbi.nih.gov/health-topics/study-quality-assessment-tools). This tool consists of a checklist of 11 items, for which an overall quality rating of “good,” “fair,” or “poor” was assigned to each study after a qualitative evaluation. Two authors independently screened the papers and performed data extraction and quality assessment. All discrepancies were settled after review of the corresponding papers by both and consensus discussions.

### 2.5 Data analysis

Statistical analyses were performed using Stata (version 14, StataCorp., College Station, TX). Random-effects models with inverse-variance method were used to estimate the pooled effect size. The Hedges g statistic was adopted as a measure of effect size based on standardized mean differences, as it is robust to small sample sizes of individual studies. The heterogeneity of results across studies was assessed by the *I*^2^ statistic and *Q* test in each analysis. A funnel plot was created to graphically identify publication bias in each meta-analysis with at least 10 studies, followed by an Egger test of asymmetry. To examine the robustness of the main findings, sensitivity analyses were performed by excluding studies without age/sex matching for controls, studies involving comorbid or medicated participants that may cause confounding bias, or studies rated as poor quality.

## 3 Discussion

Most studies focusing on HPA axis and cortisol in patients with AD have measured plasma or salivary cortisol levels.^[13]^ The assessment of CSF cortisol, however, may better reflect the cortisol concentrations to which the brain structures are exposed. In blood, the unbound, biologically active form of cortisol represents only a minor part of the total plasma levels, whereas in the CSF, cortisol is for the most part unbound. Moreover, after stimulation of the HPA axis, CSF cortisol increases rapidly and remains longer at higher levels than plasma cortisol.^[14]^

High levels of cortisol in early AD may have particularly deleterious effects on affected brain structures, contribute to the pathophysiological process, and accelerate clinical disease progression. Prolonged exposure to high glucocorticoid levels impairs cognitive function and increases the vulnerability of cerebral, in particular of hippocampal neurons for toxic events.^[15]^ Furthermore, exposure to high glucocorticoid levels may contribute to the early development of Alzheimer pathology and related cognitive impairment.^[16,17]^ In future studies, longer follow-up in larger samples of participants with mild cognitive impairment may reveal further long-term consequences of high cortisol levels. If confirmed, our study would support approaches for early detection of HPA-axis dysfunction and for interventions to counteract effects of increased cortisol levels at the predementia AD stages.

## Author contributions

Bin Liu collect data;

Xiaohong Dong designed the protocol;

Yueyang Wang performed data analysis;

Zhuang Yao finished the protocol.

**Conceptualization:** Bin Liu.

**Funding acquisition:** Xiaohong Dong.

**Investigation:** Yueyang Wang.

**Writing – original draft:** Zhuang Yao.
